# Prothrombin complex concentrate versus placebo, no intervention, or other interventions in critically bleeding patients associated with oral anticoagulant administration: a protocol for a systematic review of randomised clinical trials with meta-analysis and trial sequential analysis

**DOI:** 10.1186/s13643-018-0838-y

**Published:** 2018-10-20

**Authors:** Christian Ovesen, Jan Purrucker, Christian Gluud, Janus Christian Jakobsen, Hanne Christensen, Thorsten Steiner

**Affiliations:** 1 Department of Neurology, Bispebjerg Hospital, University of Copenhagen, Nielsine Nielsensvej 6A & B, DK-2400 Copenhagen, Denmark; 2 Copenhagen Trial Unit, Centre for Clinical Intervention Research, Rigshospitalet, Copenhagen University Hospital, Copenhagen, Denmark; 3 Department of Neurology, Heidelberg University Hospital, Heidelberg, Germany; 4 Department of Cardiology, Holbæk Hospital, Holbæk, Denmark; 5 Department of Neurology, Klinikum Frankfurt Höchst, Frankfurt, Germany

**Keywords:** Critical bleeding, Oral anticoagulation, Prothrombin complex concentrate, Intracranial haemorrhage

## Abstract

**Background:**

Acute critical bleeding is one of the most feared complications during treatment with oral anticoagulating agents. As more patients undergo treatment with anticoagulating agents, critically bleeding episodes in patients with vitamin K antagonists, thrombin inhibitor, or factor Xa inhibitor-inducted coagulopathy will be encountered frequently by physicians. Hence, an effective treatment capable of reversing the iatrogenic coagulopathy in the acute setting is needed. In randomised clinical trials and observational studies, prothrombin complex concentrate has been reported to be superior to other acute interventions, and many guidelines recommend prothrombin complex concentrate in treatment of critically bleeding patients. The aim of this systematic review is to synthesise the evidence of the effects of prothrombin complex concentrate compared with placebo, no intervention, or other treatment options in critically bleeding patients treated with oral anticoagulants.

**Methods/design:**

A comprehensive search for relevant published literature will be undertaken in Cochrane Central Register of Controlled Trials, MEDLINE, Embase, WHO International Clinical Trials Registry Platform, Science Citation Index, regulatory databases, and trial registers. We will include randomised clinical trials comparing prothrombin complex concentrate versus placebo, no intervention, or other interventions in critically bleeding patients with oral anticoagulant-induced coagulopathy. Data extraction and risk of bias assessment will be handled by two independent review authors. Meta-analysis will be performed as recommended by Cochrane Handbook for Systematic Reviews of Interventions, bias will be assessed with domains, and trial sequential analysis will be conducted to control random errors. Certainty will be assessed by GRADE.

**Discussion:**

As critical bleeding in patients treated with oral anticoagulants is an increasing problem, an up-to-date systematic review evaluating the benefits and harms of prothrombin complex concentrate is urgently needed. It is the hope that this review will be able to guide best practice in treatment and clinical research of these critically bleeding patients.

**Systematic review registration:**

PROSPERO CRD42018084371

**Electronic supplementary material:**

The online version of this article (10.1186/s13643-018-0838-y) contains supplementary material, which is available to authorized users.

## Background

The drug class of oral anticoagulants consists of vitamin K antagonists or non-vitamin K antagonists.

Vitamin K antagonists (e.g. warfarin) are a well-known class of anticoagulants prescribed for a wide range of conditions related to risk of thrombosis or emboli. It affects the coagulation cascade by inhibiting the enzyme vitamin K epoxide reductase preventing the synthesis of the biologically active forms of the clotting-factors II, VII, IX, and X [1].

Patients treated with vitamin K antagonists are at a relative high risk of haemorrhagic complications related to the iatrogenic coagulopathy. Recent results from a large Swedish registry reported an annual incidence of any haemorrhagic complication of 2.23% (95% CI 2.11 to 2.34%) [2]. One of the most dreaded complications to vitamin K antagonists is intracranial haemorrhage. The annual incidence of intracranial haemorrhagic in the previous mentioned study was reported to 0.44% (95% CI 0.39 to 0.49%). However, it is relatively well-accepted that the risk of haemorrhagic complications can be significantly mitigated by maintaining international normalised ratio (INR) within the desired range [2–4].

Non-vitamin K antagonist oral anticoagulants (NOAC) have gained increasing popularity in recent years [5–7]. In at least three Cochrane reviews, NOACs showed non-inferiority compared with warfarin [7–10]. A network meta-analysis claimed superiority of NOAC over warfarin, when adding together the net benefits of protection of embolic events and less bleeding in patients with atrial fibrillation [6].

Two general classes of NOAC exist: direct thrombin (factor IIa) inhibitors (e.g. dabigatran) and factor Xa inhibitors (rivaroxaban, apixaban, betrixaban, and edoxaban). Now, factor XI inhibitors are on the way – and we expect larger trials on these soon [11, 12]. The direct thrombin inhibitor dabigatran functions by competitive inhibition of the active site on the thrombin molecule [13]. The factor Xa inhibitors inhibit the coagulation cascade by competitively binding to the active site of factor Xa [13].

### Haemorrhagic complications

When patients on oral anticoagulants present with critical bleeding, the ability to reverse the iatrogenic coagulopathy in the acute hospital setting is important. Studies have shown that patients with vitamin K antagonists-associated intracerebral haemorrhage suffer larger admission haematoma volumes and have a greater tendency to undergo post-admission haematoma expansion compared to patients not treated with oral anticoagulants [14–17]. Post-admission haematoma expansion is consistently linked to neurological deterioration and poor functional outcome [18–21] and hence an important mediator in the association between anticoagulation treatment and poor outcome in intracerebral haemorrhage patients. A fast and efficient method to reverse the coagulopathy is warranted, if such a methods exists,as the probability of haematoma expansion is linked directly to the time spend before reversal of the coagulopathy is achieved [22].

Meta-analysis of randomised clinical trials investigating NOAC versus vitamin K antagonists for various thrombotic indications finds the risk of bleeding episodes significantly lower among patients treated with NOAC (pooled odds ratio 0.36 (95% CI 0.15 to 0.84)) [23]. This notion is supported by a recent network meta-analysis for several of the individual NOACs [6]. Patients treated with NOAC have in small observational studies been found to present with smaller intracerebral haematomas on admission compared to patients pre-treated with warfarin [24–27], however, haematoma expansion seems to happen almost as frequently [28, 29]. It is possible that these observational studies could suffer from confounding by indication and that temporal changes in treatment-guidelines could skew the results in favour of NOAC. A meta-analysis of randomised clinical trial data has not been able to show a difference in clinical outcome of patients suffering intracerebral haemorrhage, while being treated with NOAC compared with vitamin K antagonists [30].

### Reversal strategies (experimental interventions)

Different reversal strategies have been suggested and employed in the clinical setting, when treating vitamin K antagonists associated bleeding: stopping the vitamin K antagonist therapy, administration of phytomenadion (vitamin K), administration of fresh frozen plasma, or administration of prothrombin complex concentrate [31, 32]. Simply stopping the therapy with vitamin K antagonists will cause normalisation of the coagulation cascade over the course of days dependent on especially the weekly warfarin dose and the baseline international normalised ratio [33, 34]. The normalisation of the coagulation cascade is a function of the half-life of warfarin (approximately 35 to 45 h) and the synthesis of coagulation factors in the liver [34]. Phytomenadion as monotherapy has in both observational studies and randomised trials been shown effective in controlling excessive anticoagulantion (excessively raced INR) in non-critically bleeding patients [35–37], but to our knowledge, it has never been tested as monotherapy in critically bleeding patients. In randomised clinical trials, it has never been shown that factor replacement with either fresh frozen plasma or prothrombin complex concentrate is superior in terms of limiting morbidity and mortality to simply stopping the vitamin K antagonist therapy or administering phytomenadion alone. Guidelines have, however, consistently recommended using either fresh frozen plasma or prothrombin complex concentrate in addition to stopping the therapy, when treating critically bleeding patients treated with vitamin K antagonists, as replacement of the coagulation factors is deemed necessary in order to achieve an effective haemostasis [38, 39]. Many sources do recommend combining the administration of coagulation factors with phytomenadion, when treating critically bleeding patients on vitamin K antagonists. This is due to an observed re-increase in INR after 12 to 24 h in patients treated with coagulation factor replacement alone [40]. For a long time, one of the most commonly accepted reversal strategies used, when patients suffered serious haemorrhagic complications to vitamin K antagonists therapy, has been the administration of fresh frozen plasma collected from healthy donors [38]. Fresh frozen plasma replaces the coagulation factors that have been pharmacologically depleted from the vitamin K antagonists treated patients’ coagulation cascade. However, rapid administration of fresh frozen plasma might be hampered by blood type matching, maximal acceptable administration pace, and possible complications related to fluid overload [41].

During recent years, the use of prothrombin complex concentrate has come into use in critically bleeding patients. Prothrombin complex concentrate contains a collection of either three coagulation factors (II, IX, and X) or four coagulation factors (II, VII, IX, and X) in a concentration approximately 25 times higher than normal plasma [31]. Prothrombin complex concentrate might be a more attractive alternative compared with fresh frozen plasma. Prothombin complex concentrate does not require blood type matching, a lower volume is required making the risk of fluid overload smaller, and the risk of infections might be lower as prothrombin complex concentrate undergoes viral inactivation procedures [31, 42]. Observational [43–48] and randomised [49–51] evidence indicates that prothrombin complex concentrate might reverse the raised INR quicker compared to fresh frozen plasma. This can possibly be translated into a lower morbidity and mortality in patients treated with prothrombin complex concentrate.

In NOAC-treated patients, one of the most persistent concerns to clinicians has been the lack of an effective technique allowing a swift reversal of the iatrogenic coagulopathy in case of critical bleeding episodes. Eerenberg et al. [52] showed that a relatively effective biochemical reversal of the coagulopathy inflicted on healthy volunteers by pre-treatment with the factor Xa inhibitor rivaroxaban was possible by administrating prothrombin complex concentrate. Healthy volunteers pre-treated with dabigatran did, however, not obtain the same reversal effect. Boehringer Ingelheim (Germany) has released a monoclonal antibody fragment—idarucizumab—which has been shown effective at normalising the utilised coagulation assay (diluted thrombin time and ecarin clotting time) in critically bleeding patients pre-treated with dabigatran [53, 54]. Idarucizumab is now marketed and can be used in clinical practice.

Other biological antidotes proposed are andexanet alfa (Portola Pharmaceuticals, CA, USA) and ciraparantag (Perosphere, CT, USA) [55]. Andexanet alfa has been shown effective in normalising the utilised coagulation assay (chromogenic anti-factor Xa assay) in critically bleeding patients pre-treated with factor Xa inhibitors [56] even though some rebound of the anticoagulating effect seems to happen after end-of-infusion [56, 57]. Andexanet alfa has recently been approved by the United States Food and Drug Administration (media release, May 2018, http://www.portola.com). Ciraparantag (PER977) is a synthetic molecule that binds factor Xa inhibitors, direct thrombin inhibitors, and unfractionated as well as low molecular weight heparins [55]. Even though not proven effective in a critically bleeding patient population, ciraparantag has been shown effective in normalising the utilised coagulation assay (whole blood clotting time) in healthy volunteers pre-treated with endoxaban [58]. In addition to reversal of NOAC using biological antidotes, a trial is currently being conducted aiming at investigating, if tranexamic acid can limit haematoma growth in patients, who develop intracerebral haemorrhage while being treated with NOAC—the TICH-NOAC trial (NCT02866838, November 2017).

To our knowledge, neither prothrombin complex concentrate, idarucizumab nor andexanet alfa have been shown effective on patient-relevant outcomes in treating NOAC-related critical bleeding. However, guidelines recommend the use of prothrombin complex concentrate in patients suffering intracerebral haemorrhage pre-treated with factor Xa inhibitors and idarucizumab in patients pre-treated with dabigatran [39].

Another important aspect to consider when administering factor replacement (fresh frozen plasma or prothrombin complex concentrate) or when using other measures to reverse anticoagulation treatment is the risk of inducing thromboembolic events. Patient populations being treated with anticoagulants are likely at an increased risk of thromboembolic complication due to the indication of anticoagulation treatment (atrial fibrillation, mechanical heart valves, etc.). By discontinuing the anticoagulation treatment, it is likely that we expose the patients to an increased risk of thromboembolic events. Moreover, the risk of thromboembolic events can presumably be aggravated even further by administration of factor replacement [59], especially if high-dose regimens are used [60].

### Why is it important to do this review?

It is essential to establish, if the apparent effect of prothrombin complex concentrate in reversing vitamin K antagonists and NOAC induced coagulopathy translates into changes in functional outcome or mortality. In addition, a synthesis of the randomised clinical trials might allow us to assess the safety profile of the different treatment options directly. A Cochrane review from 2015 [61] assessed the comparison of prothrombin complex concentrate with fresh frozen plasma. However, this review did not specifically assess the effects of prothrombin complex concentrate versus fresh frozen plasma in the subgroup of patients with intracranial haemorrhage. This complication is especially dreaded. Moreover, a preliminary search has also identified new trials since the last update of this review [51]. Another systematic review published in 2017 conducted by Brekelman et al. [62] focused on four-factor prothrombin complex concentrate and did mostly include observational studies. The systematic literature search conducted by the authors ended in August 2015 before publication of the INR normalisation in patients with coumarin-related intracranial haemorrhages trial (INCH trial) [51]. The present systematic review aims at forming the basis for evidence-based guideline recommendations for treatment of oral anticoagulant-associated critical bleeding using prothrombin complex concentrate taking bias risks (systematic errors), play of chance (random errors), and certainty of the findings into consideration.

### Objectives

The objective of this review will be to assess the beneficial and harmful effects of prothrombin complex concentrate compared with placebo, no intervention, or other interventions in critically bleeding patients treated with oral anticoagulants.

## Methods

### Types of studies

Randomised clinical trials will be considered for assessment of benefits and harms. By focusing on randomised clinical trials, we are aware that our focus will be more on benefits than on harms and that rare or late adverse events might be missed.

### Types of participants

We intend to include patients with the following characteristics:

- Critical haemorrhage from any location. We define critical bleeding as external or internal haemorrhage indicating acute reversal of the iatrogenic coagulopathy (as defined by trialists).
- Any oral anticoagulant, e.g. vitamin K antagonist or NOAC treatment at the start of the bleeding (as defined by trialists).

### Types of interventions

#### Experimental group

Prothrombin complex concentrate. We will include trials regardless of dose, type of prothrombin complex concentrate (three or four factors, inactivated or activated), and administration regime utilised.

#### Control groups

We will include trials utilising the options listed below as control intervention:

1. Placebo or no intervention.
2. Fresh frozen plasma.
3. Vitamin K or phytomenadione.
4. Other interventions (e.g. recombinant factor VII, tranexamic acid, or biological antibodies).

#### Co-interventions

Pre-specified co-interventions will be allowed, if they are planned to be administered equally in both intervention groups, e.g. phytomenadione/vitamin K. Rescue interventions will be allowed and reported.

### Outcomes

#### Primary outcomes

- All-cause mortality.
- Health-related quality of life (any valid patient-reported continuous scale used by the trialists).
- Proportion of participants with one or more serious adverse events. We will define a serious adverse event as any untoward medical occurrence that resulted in death, was life-threatening, required hospitalisation or prolongation of existing hospitalisation, or resulted in persistent or significant disability or incapacity (ICH-GCP 1997).

#### Secondary outcomes

- Poor functional outcome on the modified Rankin Scale (mRS) or the Glasgow Outcome Scale (GOS) (as defined by the trialist). If no cut-point is defined by the trialist, mRS 4–6 and GOS 1–3 will be used. If other functional outcome scale is used, they will be included, if dichotomisation is reported by the trialist.
- Proportion of participants with at least one thromboembolic event during the follow-up period (as defined by the trialist).
- Proportion of participants with allergic reaction (as defined by the trialist).
- Proportion of participants with pulmonary oedema (as defined by the trialist).

All primary and secondary outcomes will be assessed at maximum follow-up. Primary and secondary outcomes are all deemed ‘patient-important’ and ‘critical’ [63].

#### Exploratory outcomes

- INR correction not achieved within 3 h after infusion start. If this information is not available, reported INR correction between 0.5–6 h after infusion start can be included. INR correction will be defined as described in the included study. If no INR correction level is pre-defined, INR ≤ 1.2 will be used.
- Haemostatic efficacy/haematoma expansion as defined by the trialist. The assessment performed closest to 24 h after admission will be used, if multiple assessments are reported. If the trialists do not utilise a definition of haemostatic efficacy or haematoma expansion, we will utilise the definition of haemostatic efficacy proposed by Sarode et al. [50]. If trials utilise multiple definitions of haemostatic efficacy/haematoma expansion, we will use the one most identical to the haemostatic efficacy definition by Sarode et al. [50].
- Number of patients receiving ≥ 1 transfusion of packed red blood cells within the maximum follow-up period.

### Literature database searches

We will search the following literature databases using a pre-specified search strategy:

Cochrane Central Registry of Controlled Trials (CENTRAL), Ovid MEDLINE(R) Epub Ahead of Print, In-Process & Other Non-Indexed Citations, Ovid MEDLINE(R) Daily, Ovid MEDLINE and Versions(R), EMBASE (OVIDSP), WHO International Clinical Trials Registry Platform (ICTRP), and Science Citation Index (Web of Science).

All databases will be searched from the earliest available issue. Draft of search strategy is presented in Additional file 1.

We will also search company websites as well as regulatory authorities in USA, EU, India, China, Japan, and Australia-New Zealand.

The reference lists of the identified reports will be searched for possibly missed trials. Experts within the field will be asked to supply studies not identified by the search. No language restrictions will be used. The proposed search string is presented in the appendix.

### Selection of trials

Two authors will independently screen all identified reference based on title and abstract in order to identify trials potentially fulfilling the inclusion criteria (JP and CO). Disagreement will be mediated by a third author (JCJ) and solved by discussion.

The full paper of the potentially relevant references will be provided. The two authors will make a final decision towards which trials fulfil the inclusion criteria.

### Extraction of data

Data from included trials will be extracted by two independent authors (JP and CO). Disagreement will be mediated by a third author (JCJ) and solved by discussion. Data presented in graphs can be included, if they can be read off in a precise manner. If any uncertainty exists, the authors of the trial will be contacted. In the case that needed data are not presented in the article, the authors will be contacted.

The following data will be extracted from each included trial:

Overall trial data: Name of first author, publication year, duration of the trial, number of sites, number of patients randomised in each arm, main indication for treatment, age of participants, time to treatment.

Risk of bias: Besides the review authors’ overall assessment of the risk of bias within each of the below listed bias components (allocation sequence generation, allocation concealment, blinding of outcome assessors, incomplete outcome data, selective outcome reporting, vested interest bias, and other sources of bias), the review author will substantiate his/her assessment by quotes taken from the published study material or correspondence.

Treatment regime: Type of prothrombin complex concentrate (number of factors, activated/not activated), brand name, standard prothrombin complex concentrate dose/regime, standard control-treatment type/dose/regime, standard pharmacological/haematological co-interventions (criteria, product, and dose), protocol for pharmacological/haematological rescue interventions (criteria, product, and dose).

Outcomes: For all the specified outcomes, we will log; the definition of the outcome used by the trialists (including length of the follow-up period). If the outcome measure is dichotomous, the total number of patients evaluated in each treatment group and the total number of events in each treatment group are logged. If the outcome measure is continuous, the mean or median as well as standard deviation, standard error, range or confidence interval will be logged.

In trials not following the Good Clinical Practice definition of serious adverse events, the number of serious adverse events will be defined by the sum of patients experiencing untoward medical events that the two data extractors will label as fulfilling the good clinical practice definition of a serious adverse event. Differences will be resolved by mediation by a third author (JCJ).

Conclusion: The key conclusion drawn by the authors of the trial.

### Risk of bias assessment

We will utilise the bias assessment tool presented in the Cochrane Handbook for Systematic Reviews of Interventions [64]. We choose to also assess vested interest bias, as industry sponsorship has been shown to cause overestimation of the treatment effect independent of other risk of bias domains [65, 66]. Vested interests consequently impact internal validity in trials.

Numerous studies have concluded that trials at high risk of bias overestimate the beneficial treatment effects and underestimate the harmful effects [67–71]. Two reviewers will assess each included trial independent of each other. The methodology of the individual trial will be evaluated as to the risk of bias within the following domains: *Allocations sequence generation, allocation concealment, blinding of participants and treatment providers, blinding of outcome assessors, incomplete outcome data, selective outcome reporting, vested interest bias, and other bias sources.*

#### Allocation sequence generation

- Low risk of bias—the allocation sequence is generated by a computer, random number table or similar. Minimization will be considered equal to randomisation.
- Uncertain risk of bias—the trial is reported to utilise a random allocation sequence or minimization, but the method used for the allocation sequence generation was not described.
- High risk of bias—a non-random system is used to generate the allocation sequence (quasi-randomised designs). Such trials will be excluded from assessments of benefits.

#### Allocation concealment

- Low risk of bias—the allocation sequence was concealed in a way that participants or investigators could not have predicted the next treatment allocation.
- Uncertain risk of bias—the method used to conceal the allocation sequence is not described.
- High risk of bias—the allocation sequence could have been foreseen by the investigators or participants. Such trials will be excluded from assessments of benefits.

#### Blinding of participants and treatment providers

- Low risk—if the participants and the personnel are blinded to treatment allocation and this is described.
- Unclear risk—if there is insufficient information to permit judgement of ‘low risk’ or ‘high risk’.
- High risk—if blinding of participants and personnel is not performed or if allocation could be deducted, e.g. by utilising no intervention as control treatment.

#### Blinding of outcome assessors

- Low risk of bias—the outcome assessors are blinded and the method of blinding is described.
- Uncertain risk of bias—the outcome assessors are blinded and the method of blinding is not described.
- High risk of bias—the outcome assessors are not blinded.

#### Incomplete outcome data

- Low risk of bias—there are no post-randomisation drop-outs/withdrawals or if missing outcome data are balanced between intervention groups with similar reasons posted.
- Uncertain risk of bias—it is not clear, whether there are any drop-outs or withdrawals or if the reasons for these drop-outs are not clear.
- High risk of bias—the reasons for missing data are likely to be related to true outcomes, ‘as-treated’ analysis is performed, potentially inappropriate application of simple imputation, potential for patients with missing outcomes to induce clinically relevant bias.

#### Selective outcome reporting

- Low risk of bias—the trial’s protocol is available and all of the trial’s pre-specified (primary and secondary) outcomes that are of interest in the review have been reported in the pre-specified way. Alternatively, if the protocol is not available but the published report contains all expected outcomes (including pre-specified).
- Uncertain risk of bias—there is insufficient information present to assess the risk of selective outcome reporting.
- High risk of bias—not all the pre-specified outcomes are reported, the primary outcomes are changed, one or more primary outcomes were not pre-specified or if some of the important outcomes are incompletely reported.

#### Vested interest bias

- Low risk of bias—the trial is without funding or is not funded by a pharmaceutical company or medical manufacturer, who might have an interest in the obtained results.
- Uncertain risk of bias—the source of funding is not clear.
- High risk of bias—the trial is funded by a pharmaceutical company or medical manufacturer, who might have an interest in the obtained results.

#### Other sources of bias

If other sources of bias are evident, these sources will be reported—and the consequences will be discussed.

#### Overall risk of bias

We will judge trials to be at ‘overall low risk of bias’, if they are assessed as ‘low risk of bias’ in all the above domains. We will judge trials to be at ‘overall high risk of bias’, if they are assessed as having an unclear risk of bias or a high risk of bias in one or more of the above domains.

As the risk of bias can vary between different outcomes within the same trial, we will assess the domains ‘Blinding of outcome assessor’, ‘Incomplete outcome data’, and ‘Selective outcome reporting’ for each outcome result. Thus, we will be able to assess the bias risk for each outcome result in addition for each trial (overall risk of bias of each trial). We will base our primary conclusions on the outcome results of our primary outcomes with low risk of bias.

### Assessment of reporting bias

On all outcomes, we will construct funnel plots and inspect for asymmetry. If more than ten trials are included, a formal test for funnel plot asymmetry will be conducted. Visually skewed funnel or a significant test of asymmetry will be discussed. We recognised that an asymmetric funnel plot does not necessarily imply reporting bias, but can arise due to poor methodology, inadequate analysis, small study effect, or chance [72–74].

### Data synthesis

Jakobsen and colleagues’ eight-step approach [75] represents a framework detailing, how reliable data synthesis can be performed in systematic reviews with meta-analysis. Our planned data synthesis is centred around these recommendations. Details concerning the analysis process and data synthesis are presented below.

#### Meta-analysis (fixed and random effect)

We will perform data syntheses in order to obtain a pooled effect estimate of the treatment effect. We will confer the recommendations in the Cochrane Handbook for Systematic Reviews of Interventions [64]. We will both perform fixed-effect and random-effects meta-analysis [75]. Differences in pooled effect estimates obtained from the two will be discussed [75]. We will primarily consider the most conservative estimate as the primary result [75]. We will regard the most conservative estimate as the estimate closest to zero effect. If the two pooled estimates are equal, we will use the estimate with the widest CI.

In standard meta-analysis, trials presenting zero events in one or both treatment arms can produce computational difficulties. The standard method in order to deal with zero cells is to employ continuity correction by adding 0.5 to zero cells [64, 76]. Continuity correction can potentially bias results towards the null and inflate variance. If we identify trials with zero events in one or both intervention groups, a beta-binominal regression analysis will be conducted as a supplementary analysis [77–79]. If the result of the beta-binominal regression differs from the traditional meta-analysis result, we will discuss the implications thoroughly [77–79].

#### Adjusted threshold for significance

In order to avoid inflation of the type 1 error due to multiplicity, we plan to adjust the significance level used to declare statistical significance [75]. As the Bonferroni adjustment method is often considered too conservative especially with correlated outcome measures, we plan to use the pragmatic approach suggested by Jakobsen and colleagues [75]. In order to adjust the risk of type 1 errors for multiple comparisons, we divide the value by the value halfway between 1 and the number of outcome comparisons. We assess three primary outcomes and four secondary, and we will consequently use 0.05/ 4 = 0.0125 as the risk of type 1 error (alpha-level) [75]. A 10% risk of type 2 error (beta-level) will be used in all calculations [80].

#### Trial sequential analysis

Trial sequential analysis is a technique designed to reduce the risk of chance-finding (due to random error or ‘play of chance’) in meta-analysis [81–85]. Standard meta-analysis is vulnerable to spurious finding, especially if a small number of patients are included or the participants are subject to multiple reanalyses [81–85].

In the trial sequential analysis, a meta-analytic sample size calculation (required information size) is calculated based on the expected or observed event rate within the control population, the expected clinically relevant relative risk reduction inflicted by the intervention, the chosen type 1 error (alpha-level) and the power (1-beta) [83]. In addition, this required information size is adjusted by the observed diversity in the meta-analysis [86].

To deal with the problems of low power and repeated significance testing, the TSA controls the accepted significance threshold by utilising monitoring boundaries, as long as the required information size is not reached [82, 83, 85]. Consequently, the effect of the intervention needs to provide a level of evidence large enough to cross a reliably adjusted significance threshold, before the intervention is considered superior to the control treatment, if the required information size is not obtained.

In addition, an intervention cannot be considered to be non-superior to the control, until a certain required information size has been reached as the meta-analysis runs the risk of type 2 error [83]. Analogue to the monitoring boundaries, futility boundaries can be constructed. Below this threshold, the probability of a significant treatment effect becomes so low that is can be ignored. It can hence be concluded that the intervention does not possess an intervention effect that is larger than or as large as the one postulated in the sample size calculation [83].

We plan to calculate the required information sizes for each outcome. The heterogeneity-adjusted information size (APHIS) is based on an a priori defined clinically relevant relative risk reduction of 20% for prothrombin complex concentrate compared with control, and a control event proportion based on the pooled event proportion in the control group. The proposed clinically relevant relative risk reduction is in nature arbitrary, but is proposed in the Grading of Recommendations, Assessment, Development and Evaluation (GRADE) Handbook [63]. When analysing continuous outcomes, we define a clinically relevant mean difference as the observed standard deviation divided by two [87] in accordance with a distribution-based approach to clinically relevant mean differences.

For all outcomes, trial sequential analysis-adjusted confidence intervals will be reported.

#### Bayes factor

Bayes factor denotes the ratio between the probability of the observed data given the null hypothesis and the probability of the observed data given the alternative hypothesis [88, 89]. It is hence a ratio of how compatible two competing hypotheses are with the observed data and consequently how much evidence is in support of the null hypothesis versus the alternative hypothesis.

In the context of a meta-analysis, the null hypothesis represents the hypothesis that the pooled effect of the intervention is identical to the pooled effect of the control intervention (effect estimates for binary outcome measures are in reality equal to the value 1.0, and continuous outcome measures are in reality equal to 0). The alternative hypothesis is in this context defined as the effect size used, when calculating the required information size [75].

An obtained low *p* value can be misleading [75, 90]. The observed *p* value could potentially be a type 1 error, or affected by imbalance in important prognostic factors due to a low number of randomised participants [91]. If a very large treatment effect was anticipated in the calculation of the required information size, even a statistically significant, but lower pooled effect estimate can be more compatible with the null hypothesis [75, 90].

When the Bayes factor is 1.0, the amount of evidence supporting the null hypothesis and the alternative hypothesis is identical [90]. This can be interpreted as a situation, in which the obtained effect size is halfway between null effect and the hypothesised effect size [90]. When Bayes factor is larger than 1.0, the evidence is in support of the null hypothesis—and when lower than 1.0, the evidence is in support of the alternative hypothesis.

We plan to calculate Bayes factor for all outcomes and use a Bayes factor less than 0.1 as a threshold for significance [75].

#### Missing data

If data needed are not available in the publications spawned from the trial, the authors will be contacted and the missing data will be requested.

Missing outcome data can potentially bias the effect estimates in a trial and in a systematic review [92]. If data are missing completely at random, the exclusions will not bias the effect estimate [93]. However, situations in which data can be said to be missing completely at random are rare. In most situations, missing outcome assessments are informatively missing—i.e. the probability that an outcome is missing is related to the unseen outcome per se [93]. An analysis not taking this into account runs the risk of bias.

If standard deviations of continuous outcomes are not reported in the trial and cannot be retrieved, they will be sought calculated from trial data. Is this calculation impossible, the standard deviation will be imputed from similar trials.

To assess the potential impact of the missing outcome data for dichotomous outcomes, we plan to perform the two following sensitivity analyses [75, 93].

- ‘Best-worst-case’ scenario: We will assume that the outcome of all participants lost to follow-up will favour the intervention in question, i.e. all lost to follow-up in the experimental group have survived, have had no serious adverse event, and suffered no morbidity (for all dichotomous outcomes); and all those participants with missing outcomes in the control group have not survived, have had a serious adverse event, and suffered morbidity (for all dichotomous outcomes).
- ‘Worst-best-case’ scenario: We will assume that all participants lost to follow-up will favour the control, i.e. all lost to follow-up in the experimental group did not survive, had a serious adverse event, and suffered morbidity (for all dichotomous outcomes); and that all those participants lost to follow-up in the control group had survived, had no serious adverse event, and suffered morbidity (for all dichotomous outcomes).

When analysing continuous outcomes, a ‘beneficial outcome’ will be the group mean plus two SDs (we will secondly use one SD in another analysis) of the group mean, and a ‘harmful outcome’ will be the group mean minus two SDs (we will secondly use one SD in another analysis) of the group mean [75].

We will present results from all scenarios in our review.

#### Subgroup analyses

We plan to conduct the following subgroup analysis on the chosen outcome measures:

- Trials at low risk of bias will be compared to trials at high risk of bias. If no trials are at low risk of bias, trials at relative lower risk of bias will be compared to trials at high risk of bias.
- Trials using fresh frozen plasma as control treatment compared to trials using other control interventions.
- Participants falling within the strict critical bleeding definition (listed below) compared to participants that do not fall within the strict critical bleeding definition (definition based on the GUSTO [94] and CURE [95] bleeding classifications).
  - A critically bleeding patient will be defined as having either:
    i. Life-threatening bleeding: Intracranial bleeding, bleeding requiring surgical intervention, bleeding that results in substantial haemodynamic compromise requiring acute treatment, or bleeding deemed life-threatening by trialists.
    ii. Major bleeding: Bleeding that causes substantial decrease in haemoglobin (level of decrease either ≥ 5 g/dL or as specified by trialists) or requires blood product transfusion.
    iii. Sensitive organ bleeding: Intraocular bleeding, intraspinal bleeding, pericardial bleeding or similar.
- Participants treated with four-factor prothrombin complex concentrate compared to participants treated with three-factor prothrombin complex concentrate.
- Participants with intracranial haemorrhage compared to participants with other types of bleeding.
- Participants treated with NOAC compared to participants treated with vitamin K antagonists.
- Participants treated with factor Xa inhibitors compared to participants treated with vitamin K antagonists.
- Participants treated with factor IIa (direct thrombin) inhibitors compared to participants treated with vitamin K antagonists.

### GRADE and summary of findings tables

The GRADE approach will be utilised to assess the quality of the body of evidence associated with each of the above listed seven patient-important primary and secondary outcomes [63]. ‘Summary of findings’ tables will be constructed using GRADEpro software (https://gradepro.org/). The GRADE approach appraises the quality of a body of evidence in order to assess the certainty in the effect estimates. The quality measures of a body of evidence considers within-study risk of bias, inconsistency of the results, indirectness of evidence, imprecision, and reporting bias [63]. We will conduct a subgroup analysis comparing pooled effect estimates between trials at low (or if no, at relative lower) risk of bias to trials at high risk of bias [96]. If no difference is detected, the summery of findings tables will be based on the overall analysis [66–70, 97, 98].

## Discussion

This systematic review will aim at synthesising the evidence on the effects of prothrombin complex concentrate in the treatment of critically bleeding patients treated with oral anticoagulants. Intracranial bleeding is in general believed to be one of the most feared complications of treatment with anticoagulation agents. It is also the aim of this review to assess the evidence regarding the treatment effect of prothrombin complex concentrate in patients with intracranial bleeding.

This protocol has a number of strengths. The predefined methodology is based on the Cochrane Handbook for Systematic Reviews of Interventions [64], the eight-step assessment suggested by Jakobsen and colleagues [75], trial sequential analysis [81, 82, 85], and GRADE assessment [99]. Hence, this protocol takes into account both risks of systematic errors and risk of random errors as well as the quality of the evidence.

Our protocol also has a number of limitations. We plan to include trials randomising different patient populations, and it must consequently be expected that we will include patients with different sources of bleeding as well as patients with different severity of bleeding [100].These factors may introduce heterogeneity, which will be sought investigated in subgroup analysis of the different patient populations. Another limitation is the expected use of different ways to adjudicate the same outcome measurements between trials (e.g. different scales to assess functional outcome) along with an expected variability in follow-up time. As we only include randomised trials, rare or late important safety events might be underreported [101, 102]. A final limitation is that we were aware of both previous reviews and also previous trials assessing the effects of prothrombin complex concentrate, which might result in data-driven methodology.

A Cochrane review [61] has assessed the evidence of the effects of prothrombin complex concentrate in patients treated with vitamin K antagonists. However, since the publication of this review, new randomised clinical trials have been conducted evaluating prothrombin complex concentrate to reverse vitamin K antagonist treatment, especially in patients with intracranial haemorrhage. In addition, non-vitamin K antagonist oral anticoagulants have become increasingly popular [5].

It is our intension in the planned systematic review to include and evaluate all randomised clinical trials conducted evaluating prothrombin complex concentrate in critically bleeding patients. We hope that this review will be able to guide best practice, when treating this patient group or planning future clinical research for this patient group.

## Additional file

Additional file 1: Draft of search strategy (DOCX 110 kb)

## Abbreviations

APHIS: heterogeneity-adjusted information size
CENTRAL: Cochrane Central Register of Controlled Trials
GOS: Glasgow Outcome Scale
GRADE: Grading of Recommendations, Assessment, Development and Evaluation
ICH-GCP: The International Council for Harmonisation of Technical Requirements for Pharmaceuticals for Human Use—Good Clinical Practice
INR: International normalised ratio
mRS: Modified Rankin Scale
NOAC: Non-vitamin K antagonist oral anticoagulant
SD: Standard deviation
TSA: Trial sequential analysis
WHO ICTRP: World Health Organization International Clinical Trials Registry Platform
