# Supplementary material for: Prothrombin complex concentrate versus placebo, no intervention, or other interventions in critically bleeding patients associated with oral anticoagulant administration: a protocol for a systematic review of randomised clinical trials with meta-analysis and trial sequential analysis
Source: Syst Rev. 2018 Oct 20;7:169. doi: 10.1186/s13643-018-0838-y (PMC6195723; doi:10.1186/s13643-018-0838-y)
Supplement: Supplementary file 1 — Draft of search strategy (DOCX 110 kb) [file 13643_2018_838_MOESM1_ESM.docx]

ADDITIONAL FILE 1: DRAFT OF SEARCH STRATEGY

**MEDLINE SEARCH PROFILE (Ovid MEDLINE(R) EPUB ahead of print, in-process or other non-indexed citations, Ovid MEDLINE(R) Daily, Ovid MEDLINE and Versions(R).**

**P: Patients with critical bleeding or need for urgent intervention who are on VKA.**

1. Exp Anticoagulants/
2. Exp. Vitamin K/
3. (vitamin K antagonist$ or vitamin-k$ or vitamin$ or VKA or warfarin or coumarin).tw
4. (non-vitamin K oral anticoagulant$ or NOAC$ or new oral anticoagulant$ or novel oral anticoagulant$ or DOAC$ or direct oral anticoagulant$ or TSOAC$ or target-specific oral anticoagulant$ or ODI$ or oral direct inhibitor$ or SODA$ or specific oral direct anticoagulant$).tw
5. (factor Xa$).tw
6. (thrombin inhibitor$).tw
7. (Rivaroxaban$ or Apixaban$ or edoxaban$ or betrixaban$ or $xaban$ or dabigatran$).tw
8. 1 or 2 or 3 or 4 or 5 or 6 or 7

**------------------------------**

**I: Does PCC**

1. Exp. Hemostatics/
2. (Prothrombin complex concentrate).tw
3. (Prothrombin 6adj concentrate).tw
4. (Prothrombin 6adj complex).tw
5. (PCC$ or 4F-PCC$ or 3F-PCC$).tw
6. (Kaskadil or Beriplex P/N or Beriplex or Cofact or Confidex or Kcentra or Ocplex or Octaplex or PPSB S.D or PPSB or PPSB-HT Nichiyaku or PPSB-HT or Profilnine or Profiline SD or ProthoRAAS or Prothrombin Complex Octapharm or Prothrombinex-HT or Prothrombinex or Prothromplex or Prothromplex Immuno Tim4 or Prothromplex NF or Prothromplex Total or Prothromplex Total NF or Protromplex TIM 3 or Proplex or Proplex-T or Pushu Laishi or TachoSil or Uman or Uman Complex or Uman-Complex or Uman-Complex D. I).tw
7. 9 or 10 or 11 or 12 or 13 or 14

**--------------------------------------**

**C: Compare to other intervention**

1. 8 and 15

**--------------------------------------**

**Randomized trials filter (cochrane high sensitive search strategy):**

1. Randomized controlled trial.pt.
2. controlled clinical trial.pt.
3. randomized.ab.
4. placebo.ab.
5. drug therapy.fs.
6. randomly.ab.
7. trial.ab.
8. groups.ab.
9. 17 or 18 or 19 or 20 or 21 or 22 or 23 or 24
10. 16 and 25

**EMBASE SEARCH PROFILE (Ovid Embase 1974 to present)**

**P: Patients with major bleeding or need for urgent intervention who are on VKA.**

1. Exp. Vitamin K/
2. Exp. Warfarin/
3. (vitamin K antagonist$ or vitamin-k$ or vitamin$ or VKA or warfarin or coumarin).tw
4. Exp. Anticoagulant agent/
5. (non-vitamin K oral anticoagulant$ or NOAC$ or new oral anticoagulant$ or novel oral anticoagulant$ or DOAC$ or direct oral anticoagulant$ or TSOAC$ or target-specific oral anticoagulant$ or ODI$ or oral direct inhibitor$ or SODA$ or specific oral direct anticoagulant$).tw
6. (factor Xa$).tw
7. (thrombin inhibitor$).tw
8. (rivaroxaban$ or apixaban$ or edoxaban$ or betrixaban$ or $xaban$ or dabigatran$).tw
9. 1 or 2 or 3 or 4 or 5 or 6 or 7 or 8

**------------------------------**

**I: Does PCC**

1. Exp. Blood clotting factors/
2. exp. prothrombin complex/
3. (Prothrombin complex concentrate).tw
4. (Prothrombin 6adj concentrate).tw
5. (Prothrombin 6adj complex).tw
6. (PCC$ or 4F-PCC$ or 3F-PCC$).tw
7. (Kaskadil or Beriplex or Beriplex or Cofact or Confidex or Kcentra or Ocplex or Octaplex or PPSB S.D or PPSB or PPSB-HT Nichiyaku or PPSB-HT or Profilnine or Profiline SD or ProthoRAAS or Prothrombin Complex Octapharm or Prothrombinex-HT or Prothrombinex or Prothromplex or Prothromplex Immuno Tim4 or Prothromplex NF or Prothromplex Total or Prothromplex Total NF or Protromplex TIM 3 or Proplex or Proplex-T or Pushu Laishi or TachoSil or Uman or Uman Complex or Uman-Complex or Uman-Complex).tw
8. 10 or 11 or 12 or 13 or 14 or 15 or 16

**--------------------------------------**

**C: Compared to other interventions**

1. 9 and 17

**--------------------------------------**

**Randomized trials filter**

1. Exp. controlled clinical trial/
2. Exp. randomized controlled trial/
3. randomi?ed.tw.
4. placebo.ab.
5. randomly.ab.
6. trial.ab.
7. 22 or 23 or 24 or 25 or 26 or 27
8. 18 and 25

**CENTRAL SEARCH PROFILE (Cochrane Central Registry of Controlled Trials (CENTRAL))**

1. MeSH descriptor: (Vitamin K) explode all trees
2. MeSH descriptor: (anticoagulants) explode all trees
3. vitamin K antagonist* or vitamin* or VKA or warfarin or coumarin:ti.ab.kw
4. non-vitamin K oral anticoagulant* or NOAC* or novel oral anticoagulant* or new oral anticoagulant* or DOAC* or direct oral anticoagulant* or TSOAC* or target-specific oral anticoagulant* or ODI* or oral direct inhibitor* or SODA* or specific oral direct anticoagulant*:ti.ab.kw
5. Factor Xa*:ti.ab.kw
6. Thrombin inhibitor*:ti.ab.kw
7. Rivaroxaban* or apixaban* or edoxaban* or betrixaban* or *xaban* or dabigatran*).tw
8. #1 OR #2 OR #3 OR #4 OR #5 OR #6 OR #7
9. MeSH descriptor: (Hemostatics) explode all trees
10. Prothrombin complex concentrate:ti.ab.kw
11. Prothrombin NEAR concentrate:ti.ab.kw
12. Prothrombin NEAR complex:ti.ab.kw
13. *PCC*:ti,ab,kw
14. Kaskadil or Beriplex or Cofact or Confidex or Kcentra or Ocplex or Octaplex or PPSB or PPSB-HT Nichiyaku or Profilnine or Profiline or ProthoRAAS or Prothrombin Complex Octapharm or Prothrombinex-HT or Prothrombinex or Prothromplex or Prothromplex Immuno Tim4 or Prothromplex or Prothromplex Total or Prothromplex Total NF or Protromplex TIM 3 or Proplex or Proplex-T or Pushu Laishi or TachoSil or Uman or Uman Complex or Uman-Complex:ti,ab,kw
15. #9 OR #10 OR #11 OR #12 OR #13 OR #14
16. #8 AND #15

**SCIENCE CITATION INDEX EXPANDED (web of science)**

1. TS=(vitamin* OR VKA* OR warfarin OR coumarin)
2. TS=( non-vitamin K oral anticoagulant* or NOAC* or novel oral anticoagulant* or new oral anticoagulant* or DOAC* or direct oral anticoagulant* or TSOAC* or target-specific oral anticoagulant* or ODI* or oral direct inhibitor* or SODA* or specific oral direct anticoagulant*)
3. TS=(Factor Xa*)
4. TS=(Thrombin inhibitor*)
5. TS=(Rivaroxaban* or Apixaban* or edoxaban* or *xaban* or dabigatran*)
6. #1 OR #2 OR #3 OR #4 OR #5
7. TS=(prothrombin complex concentrate OR *PCC*)
8. TS=(Kaskadil or Beriplex or Cofact or Confidex or Kcentra or Ocplex or Octaplex or PPSB or PPSB-HT Nichiyaku or Profilnine or Profiline or ProthoRAAS or Prothrombin Complex Octapharm or Prothrombinex-HT or Prothrombinex or Prothromplex or Prothromplex Immuno Tim4 or Prothromplex or Prothromplex Total or Prothromplex Total NF or Protromplex TIM 3 or Proplex or Proplex-T or Pushu Laishi or TachoSil or Uman or Uman Complex or Uman-Complex)
9. #7 OR #8
10. #6 AND #9
